# National Sisterhood of Liberal Women

**Published:** 1890-02

**Authors:** 


HALL'S
JOURNAL OF HEALTH

TRUTH DEMANDS NO SACRIFICE; ERROR CAN MAKE NONE.

VOL. 37. FEBRUARY, 1890. NO. 2.

### NATIONAL SISTERHOOD OF LIBERAL WOMEN.

A distinguished author has denominated the present as the "woman's century," and certainly if we are to measure her advancement from comparative inertia in the past into all the foremost avenues of human activity in the present, we must concede the aptness of the appellation.

The world's progress from a state of comparative savagery to its highest achievement of civilization may be measured step by step by the place assigned to and the duties required of woman, whether maid helpmate or wife.

Even in our day, among certain nations and peoples accounted civilized, the wife is held in almost utter subjection to the will of the husband, whose assumed superiority is conceded in all public or private affairs.

It is only among the most enlightened peoples of the present age that woman has been able to assert and maintain a position of almost absolute equality and independence. She is no longer relegated to the drawing-room and kitchen, nor are her duties confined to strictly indoor pursuits. All the learned professions and all pursuits requiring skill and delicacy of handling have made way for her, where her keen intellect and acute perception have placed her well in the advance. Not even in statecraft and politics—those hitherto exclusively male preroga-tives—is she content to be set aside as ineligible. Her interest and activ-ity, in all that concerns the common weal, are everywhere manifest. Naturally and humanely she is not only a fearless advocate of total abstinence, but sometimes, in defense of house and home, she becomes a raider and despoiler of groggeries and rum-shops.

We now have women authors, lawyers, doctors, preachers, lecturers, professors, journalists, editors, publishers, reporters, merchants, compositors, stenographers, brokers, dentists, farmers and storekeepers. One enterprising member of a woman's New York tea house visited, without escort, the tea districts of China and Japan, venturing far into the interior, and establishing valuable business connections, and at this writing, two women journalists are making a holiday excursion around the world, and it is safe to predict that the journey will be accomplished in much less time than ever before.

Women artists are nothing new. In this worthy field, where genius has the fullest range, she has long held a high rank. Quite recently a well-known painting of a distinguished French woman artist, sold by auction in New York City, brought the sum of fifty five thousand dollars; and for many months the gallery of Mrs. E. J. Lakey, on Fifth Avenue, was wholly furnished by the paintings of this eminent American artist mostly produced during a prolonged residence in Florence, Paris and London.

Naturally and humanely woman is the advocate of every reformatory measure of value to human kind. There are woman clubs and associations —social, charitable, religious and secular, without number, and everywhere throughout the range of the civil state are her energies exercised for the bettering of society, and, more than all, the Home.

The women of America have recently set on foot a "National Organization of Liberal Women," which it is believed is destined to exert a widespread influence for good; for, in all history we can point to no instance of organized womanly endeavor, whose object was not both laudable and beneficial. Let woman have her way and the world will be the better for it.

